# Fluorescently Labeled α-Conotoxin TxID, a New Probe for α3β4 Neuronal Nicotinic Acetylcholine Receptors

**DOI:** 10.3390/md20080511

**Published:** 2022-08-12

**Authors:** Meiling Huang, Xiaopeng Zhu, Yishuai Yang, Yao Tan, Sulan Luo, Dongting Zhangsun

**Affiliations:** 1 Key Laboratory of Tropical Biological Resources of Ministry of Education, School of Life Sciences, Hainan University, Haikou 570228, China; 2 Medical School, Guangxi University, Nanning 530004, China

**Keywords:** α-Conotoxin TxID, α3β4 nAChR, fluorescent probe, two-electrode voltage clamp, application

## Abstract

Neuronal nicotinic acetylcholine receptors (nAChRs) are important ion channel membrane proteins that are widely distributed in the central nervous system (CNS) and peripheral nervous system (PNS). As an important member, α3β4 nAChRs are related to pain sensation in PNS and nicotine addiction in CNS. However, research related to the α3β4 nAChRs is greatly limited by the lack of subtype-selective pharmacological tools. The α-conotoxin (α-CTx) TxID from the marine cone snail, *Conus textile*, is a selective α3β4 nAChR antagonist with relatively high potency. In this study, a fluorescent dye (5-TAMRA SE) was used to label TxID on the N-terminus of α-CTx TxID, and pure TxID-F (fluorescent analogue of TxID) was obtained by HPLC. At the same time, the potency and selectivity of TxID-F were detected by high-performance liquid chromatography (HPLC). Additionally, the potency and selectivity of TxID-F were determined by using a two-electrode voltage-clamp technique on various nAChRs expressed in the *Xenopus* oocyte expression system. The results obtained by electrophysiology showed that TxID-F maintained the same order of potency (IC_50_ 73 nM) as the native toxin (IC_50_ 25 nM) for the α3β4 nAChR subtype. In addition, the results of fluorescent spectroscopy and circular dichroism showed TxID-F has the same fluorescence as 5-TAMRA SE, as well as similar profiles as TxID. The results of flow cytometry showed that the histogram shifted significantly to the right for the RAW264.7 cells expressing α3β4-containing nAChRs stained with TxID-F and confirmed by live cell imaging. The study of fluorescent-labeled α-CTx TxID provides a rich pharmacological tool to explore the structure–function relationship, distribution, and ligand-binding domain of α3β4 nAChR subtype in the future.

## 1. Introduction

Nicotinic acetylcholine receptors (nAChRs) are transmembrane ligand-gated ion channels, which are widely distributed in the central and peripheral nervous systems of primitive and evolutionary advanced organisms [1,2]. As a member of the ligand-gated ion channel family, nAChRs play a vital role in regulating signal transmission between nerve and muscle [3]. All nAChR subtypes pentamers are composed of homologous subunits or heterologous complexes [4,5]. So far, there are 16 kinds of nAChR subunits identified in mammals, such as α1–α7, α9, α10, and β1–β4, as well as γ, δ, and ε [6]. It is worth noting that the nAChR subtypes have different pharmacological sensitivities, due to different subunits, but their structures are very similar and difficult to distinguish [7,8]. Neuronal-nAChR subtypes are involved in a range of disease states, including Parkinson’s disease, Alzheimer’s disease, sleep-related hypermotor epilepsy, nicotine addiction, and lung cancer [9,10,11,12,13].

In the mammalian brain, the medial habenula (MHB) expresses high nAChR levels [14,15,16]. Recently, α3-containing nAChRs present in the medial habenula have attracted substantial attention because of their potential role in influencing nicotine addiction [17,18,19]. In addition, recent studies have shown that the gene polymorphism of the α3 and β4 subunits in vivo are related to nicotine addiction and drug abuse [20]. The α3β4 nAChR subtype is mainly located in the MHB, interphalangeal nucleus (IPN), and fasciculus retroflexus (FR) of the rat brain. In addition, it is also expressed in a large number of dopamine pathways, as well as the sensory and autonomic neurons in the midbrain [16,19]. The α3β4 nAChR can mediate the release of noradrenaline (NE), and it plays an important role in nicotine reward and nicotine withdrawal syndrome [21]. Therefore, peptides inhibiting α3β4 can be used as pharmacological tools to understand the roles of α3β4 nAChRs in nicotine reward and nicotine withdrawal syndrome. It was recently reported that the selective α3β4 nAChR antagonists AT-1001 and 18-methoxycoronaridine (18-MC) can reduce nicotine self-administration in rats, demonstrating the role of α3β4 nAChR in nicotine dependence and drug-seeking behavior [22]. Although α3β4 nAChRs are involved in mediating many neurophysiological processes, the lack of tracer antagonists limits studies on their structure, function, and pharmacological activity.

Conotoxins (CTx) are small peptides from the venom of tropical mollusk cone snails [23]. Conotoxins can specifically act on ion channels and receptors, so they can be used as an important tool for neurophysiological and pharmacological research [24]. This family of α-CTx generally belongs to superfamily A, which is generally composed of 6–20 amino acids with two disulfide bonds, and most of the C-terminal amino acids are amidated [25,26]. As selective antagonists of a variety of neural and muscle nAChR subtypes, the α-CTxs have the characteristics of strong selectivity, stable structure, and low side effects [21,27,28]. Therefore, they are often used as an important probe to study the interaction between the ligand and receptor. The structure–function relationship even can be further developed as a drug lead for nAChRs function-related diseases [23]. However, α-CTxs antagonists of the α3β4 nAChR are still scarce. A-CTx TxID was identified from *Conus textile* in our laboratory earlier, which contains 15 amino acid residues with two disulfide bonds. The amino acid sequence is GCCSHPVCSAMSPIC# (# = C-terminal carboxamide), and its IC_50_ value at α3β4 nAChR is 12.5 nM, which is relatively the most potent antagonist of α3β4 nAChR so far [24].

In recent years, fluorescent labeling technology has been widely used due to its advantages of good selectivity, high sensitivity, and simple operation [29]. At present, Rhodamine is the most common dye in fluorescence labeling technology, which has the characteristics of high yield, high molar absorption coefficient, long excitation wave, and high fluorescence quantum yield [30,31]. Most importantly, the fluorescent analogue of rhodamine usually maintains a certain degree of biological activity, so they are often used to construct labeled peptides or protein fluorescent probes [29]. At present, a small amount of the literature has reported fluorescently labelled conotoxins, including α-MII, which has been connected to four different fluorophores, while RgIA-5727 has Cy3 attached [29,32]. In all cases, binding and selectivity were retained, thereby making them valuable probes to visualize nAChR subtypes. In addition, they can be used in the fields of cell imaging and flow cytometry [33,34,35]. However, there is no study on the fluorescent labeling of α-CTx antagonist targeting the α3β4 subtype.

In this study, 5-carboxytetramethyl rhodamine succinimide ester (5-TAMRA, SE) was used for labeling of α-CTx TxID, and the conjugated product TxID-F was obtained after the reaction. At the same time, the potency and selectivity of TxID-F were determined by the two-electrode voltage-clamp technique on various nAChR subtypes that are heterologously expressed in *Xenopus* oocytes. We found that the IC_50_ value against the rα3β4 nAChR subtype was comparable with that of the native α-CTx TxID, and the high selectivity of TxID was maintained. The results of fluorescence spectrum and circular dichroism showed that TxID-F has the same fluorescence as rhodamine, as well as a similar profile as TxID. The results of flow cytometry showed that the histogram shifted significantly to the right for RAW264.7 cells expressing α3β4-containing nAChRs stained with TxID-F. Red fluorescence was also observed on the cell membrane by live cell imaging. α-CTx TxID fluorescent probe provides an essential pharmacological tool that selectively targets α3β4 nAChR.

## 2. Results

### 2.1. α-CTx TxID Linear Peptide Oxidative Folding

**Figure 1:**
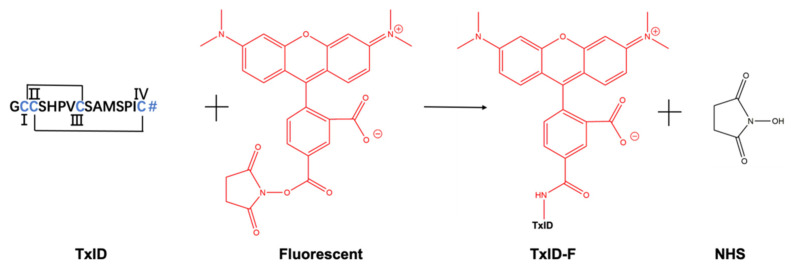
Conjugation reaction between 5-TAMRA SE and α-CTx TxID. The site coupling with 5-TAMRA SE is located at the N-terminus of α- CTx TxID. TxID contains two disulfide bridges between cysteine residues, and the N-terminal contains a free amino acid residue. # = C-terminal carboxamide. I, II, III and IV represent the positions of cysteine, respectively.

α-CTx TxID is a peptide that was determined based on the predicted sequence of a gene isolated from the vermivorous marine cone snail C. textile, and the crude TxID linear peptide was synthesized by solid-phase synthesis, as described in section “Materials and Methods”. The crude TxID linear peptide (~85% purity) was purified by preparative RP-HPLC (reversed-phase high-performance liquid chromatography), and the mass of TxID (>95% purity) was confirmed by electrospray ionization-mass spectrometry (ESI-MS); the mature TxID was obtained by the two-step oxidative folding strategy. The fluorescent reporter group was added to a high-affinity peptide ligand of α3α4 nAChR. The process of TxID-F synthesis was shown in Figure 1. There is only one free amino group available for coupling with 5-TAMRA SE by analyzing the amino acid sequence, and we firmly believe that the fluorescent group will be connected to the N-terminus of TxID, because lysine (Lys) is not present in the sequence of TxID. Furthermore, the reaction between the free amino group and SE proceeds via nucleophilic acyl substitution to form a carboxamide bond between the peptide and dye.

**Figure 2:**
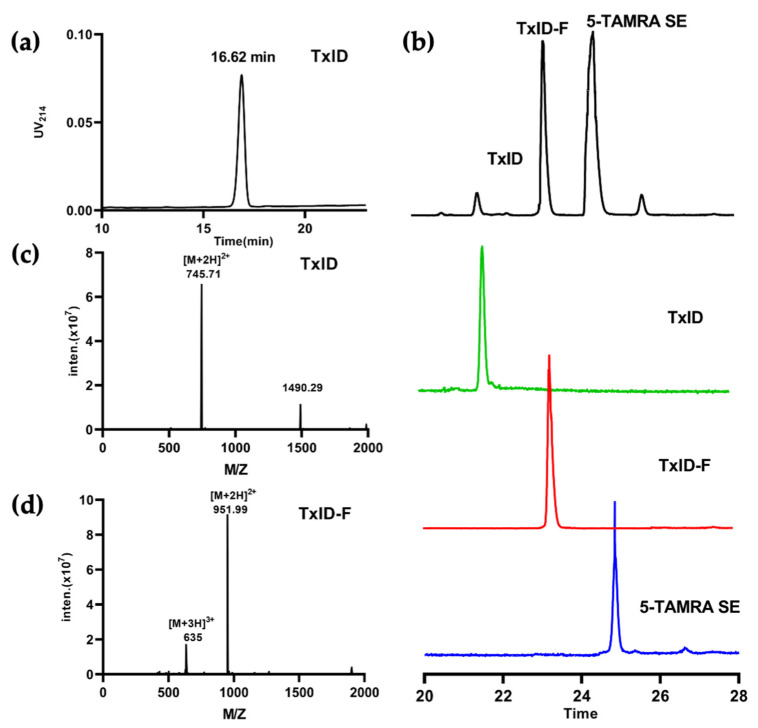
HPLC chromatograms and mass spectra of TxID and TxID-F. The peptides were analyzed on a reversed-phase analytical Vydac C18 HPLC column. Absorbance was monitored at 214 nm. (**a**) HPLC chromatogram of TxID, the retention time of TxID is 16.62 min with the linear gradient of 10% buffer B to 40% buffer B over 20 min. (**b**) HPLC chromatogram of TxID-F in the mixture of conjugation reaction; the retention time of TxID-F is 23.24 min by using a linear gradient of 5% buffer B to 55% buffer B over 35 min (TxID: green, TxID-F: red,5-TAMRA SE: blue). (**c**) Electrospray ionization mass spectrometry (ESI-MS) data for TxID with the observed monoisotopic mass of 1489.42 Da. (**d**) ESI-MS data for pure TxID-F with the observed monoisotopic masses of 1901.98 Da.

The final product, TxID-F, was separated by RP-HPLC and confirmed by ESI-MS. RP-HPLC analysis revealed that fluorescent conjugation of TxID resulted in a shift in retention time for the conjugated peptide, compared with wild-type TxID, thus indicating increased hydrophobicity (Figure 2a,b). The desired product, TxID-F, was eluted at 38.18% buffer B (buffer B was 90% ACN and 10% ddH_2_O with 0.1% TFA; buffer A was ddH_2_O with 0.1% TFA), which was earlier than that of 5-TAMRA SE (Figure 2b). TxID-F was eluted as a single peak, with a peak purity analysis in HPLC greater than 95%. The observed monoisotopic masses for TxID and TxID-F were 1489.42 and 1901.98 Da, respectively, which are consistent with the theoretical molecular weights (Figure 2c,d).

### 2.2. Fluorescence Spectrum and Circular Dichroism Analysis of TxID-F

**Figure 3:**
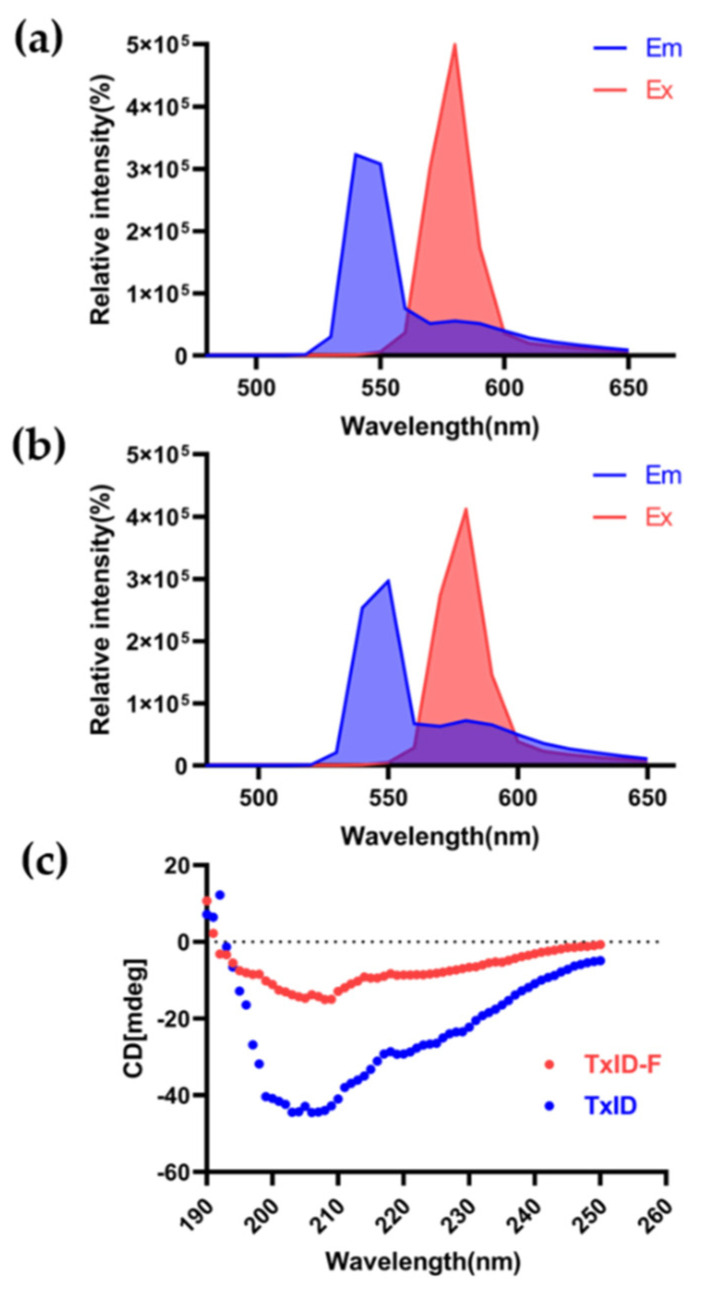
Fluorescence spectrum and circular dichroism analysis of TxID−F. Fluorescence spectrum of 5-TAMRA SE (**a**) and TxID−F (**b**). (**c**) The circular dichroism analysis (TxID: blue; TxID−F: red).

The 5-TAMRA SE dye molecule is suitable for fluorescence measurements of biological samples because it has an emission wavelength (550~650 nm) at the red end of the visible range selected. The full-wavelength scan results displayed that both TxID and TxID-F have their absorption peaks, and there was no significant change between 5-TAMRA SE and TxID-F. The fluorescence excitation and emission spectra of TxID-F and 5-TAMRA SE were quite similar (Figure 3a,b), thus indicating that TxID is labeled with the same fluorescence as 5-TAMRA SE after conjugation. Further, we investigated the conformational changes of fluorescent analogue using circular dichroism (CD). After fluorescent labeling of TxID, the results showed that, similarly, both TxID and TxID-F have two strong negative peak wavelengths around 208 and 220 nm, as well as a positive peak at 193 nm, which indicates that both TxID and TxID-F contain α-helical motifs (Figure 3c).

### 2.3. Pharmacological Activity of TxID-F

The lyophilized purified fractions (TxID-F with ~95% purity) that were confirmed by ESI-MS and tested on several different nAChR subtypes expressed in Xenopus laevis oocytes by using a two-electrode voltage clamp. 

**Figure 4:**
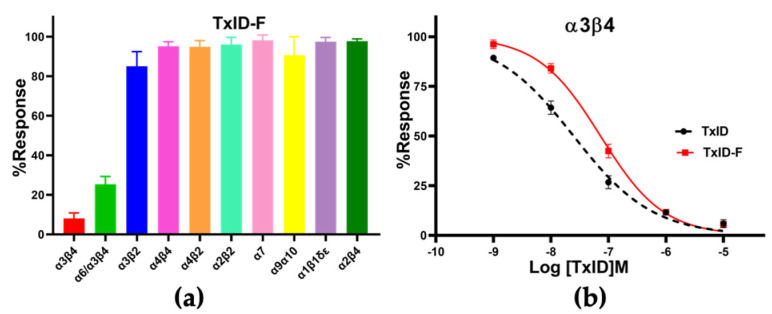
Pharmacological activity of TxID−F. (**a**) Inhibitory activity of TxID−F (10 μM) against other nAChR subtypes. (**b**) Concentration−response analysis of TxID and TxID−F potency against α3β4 nAChR subtype. The current was induced by 100 μM ACh. The IC_50_ value of TxID−F showed a 3−fold decrease, compared with WT TxID. All IC_50_ values and hill slopes were shown in Table 1. Error bars mean ± SEM. One−way ANOVA was used for comparison between different groups. All data were obtained from 3–6 separate oocytes for each experimental determination. All receptors were of rat origin, except α1β1δε, which was of mouse origin.

CTx TxID-F (10 μM) was applied to oocytes expressing nAChRs. TxID-F had the most inhibitory activity at rα3β4, followed with the rα6/α3β4 subtype, and almost no activity at the other nAChR subtypes (Figure 4 and Figure S1). Compared to WT TxID, the selectivity of the fluorescent analogue did not change significantly. In concentration–response curves, the IC_50_ values of TxID and TxID-F against α3β4 were 25 and 73 nM, respectively (Figure 4b, Table 1).

### 2.4. TxID-F Detection by the RAW264.7 Cell Line

**Figure 5:**
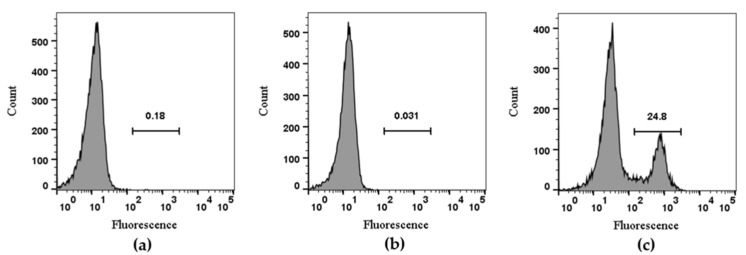
Flow cytometry analysis of TxID-F on RAW264.7 cells. Control RAW264.7 cells alone are compared using flow cytometry to the cells with TxID and TxID-F (RAW264.7 cell: (**a**), TxID: (**b**), TxID-F: (**c**). Histograms indicate live cell populations (*n* = 4).

The RAW264.7 cell line was used to evaluate TxID-F detection of α3β4-containing nAChRs; previous studies have shown that both α3 and β4 subunits are expressed on the cell membrane [36]. The experiment was divided into three groups. The blank group was RAW264.7 cells alone, the negative control group was added with conotoxin without fluorescence modification, and the experimental group was added with TxID-F. The results showed that the strong fluorescence intensity can be detected in the experimental group; according to the histogram detected by flow cytometry, compared with the blank and negative control groups, the histogram of the experimental group shifted to the right by about one order of magnitude (Figure 5). 

**Figure 6:**
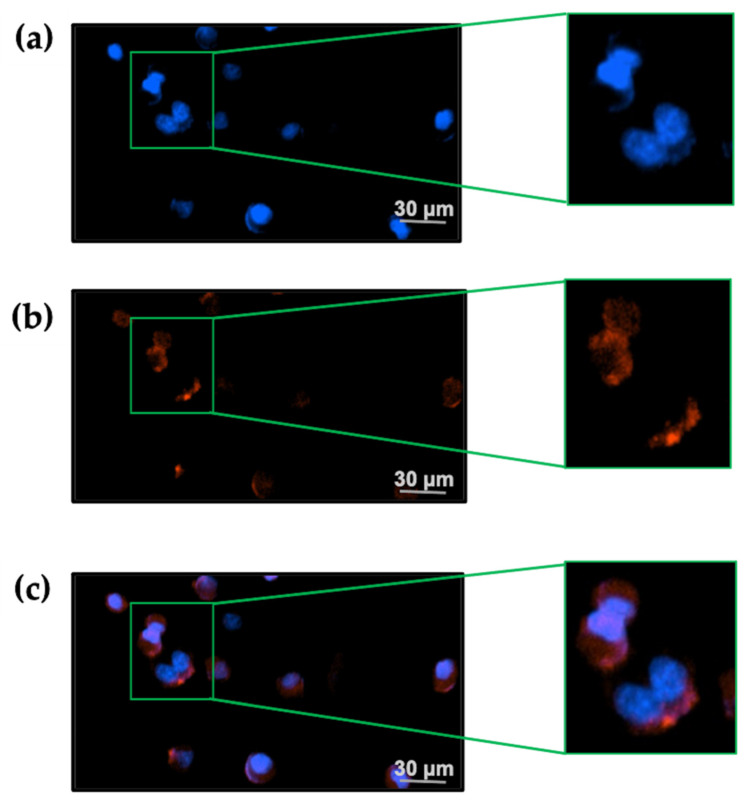
TxID-F selectively labels RAW264.7 cells expressing α3β4 nAChR. (**a**) The cells in all images were counter-stained with Hoechst 33342 (blue: 377,447 nm) to label the nucleus. (**b**) Cells were labeled with 20 nM TxID-F (red: 586, 647 nm). (**c**) All cell images were obtained by a living cell cytation1 imagine reader at 20× objective; the scale bar is 30 μm.

In the presence of 20 nM TxID-F, red fluorescence signal was observed on the RAW264.7 cells, and no fluorescence signal was detected in the control group (Figure 6), thus indicating that the TxID-F labeling of the RAW264.7 cell line represents specific binding to α3β4 nAChR.

## 3. Discussion

Venoms of marine snails of the genus *Conus* are natural combinatorial peptide libraries [37]. Different classes of conotoxins (CTxs) or conopeptides have high selectivity toward various ion channels and receptors [38]. At present, a large number of conotoxins targeting nAChR have been found from *Conus* snails that can distinguish different subtypes of the receptor. For example, TxIB (obtained from *C. textile* by our laboratory in 2013) can specifically act on α6/α3β2β3 (α6β2*) nAChRs, with an IC_50_ value of 28 nM, and it can be used as a pharmacological tool to evaluate the function and related diseases of α6/β2* nAChRs, such as treatment that affected the expression of morphine-induced conditioned place preference [21]. LvIA, isolated from *C. lividus* in 2014 by our laboratory, is a polypeptide molecule with the best selectivity and activity for α3β2 nAChR, and its potency at the human α3β2 nAChR is 305 times higher than that of α6/β2* nAChRs [38]. Other CTxs, such as GID, found by Jayati et al., showed specific selectivity for the α4β2 nAChR subtype [39]. In addition, α-CTxs ArIB can effectively act on the α7 nAChR subtype [40]. α- CTxs TxID is a small neuropeptide identified in *C. textile* that contains 15 amino acid residues and 2 disulfide bonds. At present, the selective target of the toxin has been determined and can be used for the study of neurophysiology and pharmacology, as related to nAChRs. TxID is a strong antagonist against α3β4 nAChR (IC_50_ = 12.5 nM) [27]. The previous research showed that TxID has anti-nicotine addiction effect and is expected to be developed as a new type of smoking cessation drug [21].

With the continuous development of fluorescent labeling technology, the application of polypeptide probes has been expanded [41]. At present, there are relatively few studies on fluorescent conotoxin probes; for example, Arik J. hone et al. used Cy3- and Alexa Fluor 546-labeled ArIB [V11L; V16A] to visualize the distribution of α7 nAChRs in the brain [40]. Markus Muttenthaler et al. labeled RgIA with Cy5 to observe receptor localization in mouse RAW264.7 macrophage cell lines [35]. Fernando Fisher et al. labeled RgIA-5474 with Cy3 to obtain Cy3-RgIA-5727, which is α9α10 selective and an effective antagonist; additionally, the distribution and lateral mobility of voltage-dependent Ca^2+^ channels on CA1 hippocampal neurons have been determined with biotinylated derivatives of the selective probe ω-CTx labeled by biologically active fluorescent [42]. So far, the fluorescent probe targeting α3β4 nAChR has not been reported. In this study, the fluorescent probe, obtained based on TxID, not only maintains the fluorescence activity but also retains the inhibitory activity of α3β4 nAChR, and it can be used as an important tool for the study of α3β4 nAChR structure and function.

In this study, 5-TAMRA was coupled to α-CTx TxID to construct the fluorescent conjugate TxID-F, as the NHS (N-hydroxysuccinimide) of 5-TAMRA SE can chemically bind to the free amino group in the TxID sequence. In addition, there is only one reactive site on the N-terminus of the TxID sequence, which ensures that the product obtained after the reaction is a unique substance. Further, the changes in the site where succinimide ester is labeled has little effect on the activity of the TxID. Through previous studies, we found that the change of secondary structure of conotoxin will affect α-CTx activity. The results of circular dichroism show that, although there are differences after fluorescently labeling TxID, TxID-F has a similar profile to TxID. Anyway, both TxID and TxID-F have two strong negative peak wavelengths around 208 and 220 nm, as well as a positive peak at 193 nm, which indicates that both TxID and TxID-F contain α-helical motifs. In addition, full-wavelength scanning results display that the fluorescent-labeled has fluorescence emission spectra with excitation at 546 nm; its absorption wavelength is the same as that of fluorescent dye 5-TAMRA, thus indicating that TxID-F has the properties of fluorescent dye. To further verify the biological activity of the TxID fluorescent probe, we determined its activity in vitro via the two-electrode voltage patch-clamp technique. The results showed that the IC_50_ value of TxID-F at α3β4 nAChR was 73 nM, indicating that its activity did not change. Previous studies have shown that the α3 and β4 nAChR subunits are highly expressed on RAW264.7 [36]. In this research, TxID-F was able to detect α3β4-containig nAChRs on the RAW264.7 membrane surface in both flow cytometry and cell imaging.

Finally, considering that TxID has an effect in anti-nicotine addiction, probes such as TxID-F will become highly valuable for assisting in elucidating the precise molecular target mechanisms involved. TxID was labeled by 5-TAMRA-SE; this idea will lead the way for detecting and quantifying the presence of specific α3β4 nAChR subtype on the protein level, a difficult and challenging task, due to the current lack of subtype-specific nAChR imaging probes. Moreover, the research of TxID-F detection by the RAW264.7 cell line presented here represents only a rudimentary data set, and more extensive work (that is outside of the scope of this work) is currently being developed to further define the application of such imaging probes and expand our knowledge on the distribution and function of nAChRs in different cells.

In conclusion, the linear TxID was obtained by solid-phase synthesis, and the bioactive TxID was prepared by two-step oxidation method. Then, the 5-MAMRA-SE was labeled on theTxID to product a fluorescent peptide with both fluorescent properties and bioactivity. This fluorescent peptide can be used to label α3β4 nAChR on RAW264.7 cells, and it provides an essential pharmacological tool for studying the location, function, and pharmacological activity of α3β4 nAChR.

## 4. Materials and Methods

### 4.1. Materials

Clones of mouse (m) α1, β1, δ, ε, and rat (r) α2, α3, α4, and α7, as well as β4 cDNAs, were generously provided by S. Heinemann (Salk Institute, La Jolla, CA, USA). Clones of (r) α9 and (r) α10 were kindly provided by A.B. Elgoyen (Instituto de Investigaciones en Ingeniería Genética y Biología Molecular, Buenos Aires, Argentina). rα6/α3 chimera clone was kindly provided by J.E. Garrett (Cognetix, Inc., Salt Lake City, UT, USA). C.W. Luetje (University of Miami, Miami, FL, USA) provided clones of (r) β2 and (r) β3 subunits in the high-expressing pGEMHE vector.

Mouse macrophage cell line RAW264.7 was purchased from the BNCC Cell Bank (Beijing, China). Fetal bovine serum (FBS) was purchased from Gibco Life Technologies (Rockville, MD, USA). Dulbecco’s modified Eagle’s medium (DMEM) was purchased from Gibco Life Technologies (Rockville, MD, USA). Hochest was purchased from Beyotime Biotechnology (Shanghai, China). Trypsin was purchased from Gibco Life Technologies (Rockville, MD, USA). The 5-TAMRA SE was purchased from Fanbo Biochemical (Beijing, China). Acetonitrile (ACN, HPLC grade) was purchased from Thermo Fisher Scientific (Pittsburgh, PA, USA). RNA transcription and purification kits were purchased from Thermo Fisher Scientific (Austin, TX, USA). Acetylcholine chloride and other reagents were obtained from Sigma (St. Louis, MO, USA). Analytical reversed-phase C18 Vydac columns (5 μm, 4.6 × 250 mm; 10 μm,22 × 250 mm), and a preparative C18 Vydac column (10 μm, 22 mm × 250 mm) was purchased from Grace Vydac (Hesperia, CA, USA). 

*X. laevis* was obtained from the Kunming Institute of zoology CAS (CHN). The protocol to obtain *X. laevis* oocytes was approved by the Ethics Committee of Hainan University, and we strictly adhered to the guidelines for the care and use of laboratory animals in this study. Oocytes from *X. laevis* were surgically removed and prepared as previously described [18].

### 4.2. TxID Linear Peptide Synthesis and Oxidative Folding

The crude linear peptides were successfully synthesized using Fmoc chemistry by Bankpeptide Biological Technology Co., Ltd. After a two-step oxidative folding procedure of TxID in our lab, we obtained bioactive peptides [43]. In brief, the two-step oxidation protocol was as follows: 20 mg of purified linear TxID was added to the oxidized solution (0.6 g Tris and 0.3 g potassium ferricyanide (K_3_(Fe(CN)_6_)) in 150 mL ddH_2_O, pH 7.5). Magnetic stirring was performed at 25 °C for 45 min to form the first disulfide bond between Cys I and Cys III. The monocyclic TxID was purified by reverse-phase high-performance liquid chromatography (RP-HPLC). Elution condition: 10–40% buffer B over 20 min, with a flow rate of 10 mL/min and UV detection at 214 nm. The purified monocyclic TxID was added to the oxidized solution (0.35 g I2 is dissolved in 8 mL CAN, 24 mL ddH2O, and 0.98 mL TFA). Magnetic stirring was performed at 25 °C for 15 min, and saturated VC was added to make the solution colorless. The whole reaction was carried out under nitrogen protection. The monocyclic TxID is catalyzed to form a second disulfide bond between Cys II and Cys IV. The purity and quantity of the TxID were assessed by analytical RP-HPLC. The molecular weight of the final product was identified by ESI-MS.

### 4.3. Exploration of the Method of Labeling TxID with 5-TAMRA SE

The 500 nmol TxID was dissolved in 800 μL of sodium borate buffer and reacted with 1mg of 5-TAMRA SE, which was dissolved in 200 μL of DMSO. The reaction was carried out on a shaking table at room temperature without light for 4 h, and the reaction was terminated using 400 μL 0.1% (*v/v*) TFA. The reactants were separated by preparative RP-HPLC. Elution condition was as follows: 5–55% buffer B over 35 min, with a flow rate of 10 mL/min and UV detection at 214 nm. The molecular weight of the product was identified by ESI-MS. The whole process was performed devoid of light, so as not to damage the photosensitive structure. 

### 4.4. Circular Dichroism and Fluorescence Spectra

The circular dichroism of TxID and TxID-F (10 μM peptide dissolved in phosphate buffer solution, pH 7.5) were measured by using a MOS-500 spectropolarimeter (BioLogic, Seyssinet-Pariset, France) in quartz cells of 0.1 cm pathlengths at room temperature, using the following parameters: 0.5 s response; 100 nm/min scanning speed; 1 nm data acquisition interval; standard sensitivity; 3 accumulations; 15 mL/min flow of nitrogen; and 1 nm bandwidth. The results were smoothed using the noise reduction routines provided with the J-810 spectropolarimeter. Dichro Web was used to analyze and process data. A Spectra Max M2 (Molecular Devices, San Jose, CA, USA) was employed to scan the wavelength of 5-TAMRA SE and TxID-F. Fluorescence emission spectra were recorded between 450 and 650 nm, with excitation at 546 nm; fluorescence excitation spectra were collected between 450 and 650 nm at the emission of 575 nm. 

### 4.5. Activity Assay of the Fluorescent Analogue

The nAChRs expression model was constructed in vitro with *Xenopus* oocytes expression system. The inhibiting potency of TxID and TxID-F on various subtypes of nAChR were detected by two-electrode voltage clamps (TEVC). The preparation of *Xenopus* oocytes, acquisition of nAChRs RNA, and microinjection were described previously [44]. In brief, different subtypes of cRNA were injected into *Xenopus* oocytes. It is worth noting that each subtype of cRNA was combined in equimolar ratios, and the minimum injection mass for each subunit was 10 ng. The oocytes were cultured at 17 °C, with humidity of 35% in culture medium (96 mM NaCl, 1.8 mM CaCl_2_, 1 mM MgCl_2_, 2 mM KCl, and 5 mM HEPES; 10 mg/L streptomycin, 10 mg/L penicillin, and 100 mg/L gentamicin, pH 7.0−7.5). The oocytes expressing various nAChR subtypes on the membrane surface were placed at 50 μL chamber and perfused with ND96 (96 mM NaCl, 2 mM KCl, 1.8 mM CaCl_2_, 1.0 mM MgCl_2_, 5 mM HEPES, 0.5% BSA, pH 7.5), containing 0.5% BSA, at a flow rate of 2 mL/min. The current response to ACh (100 μM) was tested using the double electrode voltage clamp amplifier Axon 900A (Molecular Device, Sunnyvale, CA, USA) at the holding potential of −70 mV. The ND96 containing 0.5% BSA was used as blank control. The ACh-induced control current was repeated three times to obtain the average peak current. There is a 1-s pulse of 100 μM ACh in one sweep corresponding to the subtypes, except α7 (200 μM ACh), α1β1δε, and α9α10 subtypes (10 μM ACh) [38]. The peak current amplitudes were recorded and analyzed by Clampfit 10.2 software (Molecular Devices Corp., Sunnyvale, CA, USA) before and after peptide incubation at 20–26 ℃. The magnitude of membrane current at each concentration (peptides were diluted to 10^−4^, 10^−5^, 10^−6^, 10^−7^, and 10^−8^, respectively) was recorded. At least 3 oocytes were tested at each concentration.

### 4.6. Detection by Flow Cytometry

The RAW264.7 cells cultured in DMEM containing 10% FBS at 37 ℃ and 5% CO_2_ were digested with trypsin without EDTA, washed twice with precooled PBS buffer, and centrifuged at 2000× *g* for 5 min. The cells were resuspended in PBS, and 20 μL PBS, 20 μL TxID, and 20 μL TxID-F were added to each group, respectively; then, they were fully mixed and reacted at room temperature and away from light for 10 min. Each group had 4 replicates. After the reaction, the precooled PBS buffer was washed 2–3 times and centrifuged at 2000× *g* for 5 min. Then, Guava easy Cyte^TM^ (Merck, CA, USA) was used for detection.

### 4.7. Fluorescence Imaging

The RAW264.7 cells were cultured and grown in DMEM containing 10% FBS at 37 ℃ and 5% CO_2_. At a cell confluence of about 80%, they were dissociated with trypsin. For the TxID-F labeling experiment, 10^5^ cells were inoculated in a six-well plate and cultured overnight. The next day, the growth medium was removed, and the cells were washed twice with PBS. Then, the TxID-F (20 nM) and Hoechst 33342 (1 mL) were applied to cells and cultured at room temperature for 20 min. Cells were washed with PBS 2–3 times to remove unbound TxID-F and excess nuclear stain. Data were collected by Cytation 1 imagine reader (BioTek, Winooski, VT, USA).

### 4.8. Data Analysis

For the baseline response, at least three ACh responses were averaged. The response of the α-CTx TxID was defined as the peak current amplitude at the Ach-induced steady-state current, and the value was divided by the toxin pre-baseline value to calculate a “% response”. Each data point of a dose-response curve represents the mean ± SEM of at least three oocytes. The dose-response data were fitted to the equation: response (%) = 100/[1 + ([toxin]/IC_50_)^Hill slope^], where hillslope was the Hill coefficient, and IC_50_ value was calculated for the concentration of antagonist producing a half-maximal inhibition using GraphPad Prism 6.0 (GraphPad Software, San Diego, CA, USA).

## Figures and Tables

**Table 1:** Potency of TxID and TxID-F at the α3β4 nAChR subtype.

Peptide	IC_50_ (nM)	Hill Slope
TxID	25 (20–32)	0.6 (0.5–0.7)
TxID-F	73 (60–89)	0.8 (0.7–0.9)

Numbers in parentheses are 95% confidence intervals (C.I).

## Data Availability

The data presented in this study are available on request from the corresponding author. The data are not publicly available due to need for further research.

## Supplementary Materials

The following supporting information can be downloaded at: https://www.mdpi.com/article/10.3390/md20080511/s1. Figure S1: Electrophysiological activity of TxID-F. The potency of TxID-F on various nAChR subtypes. α3β4 (a), α6/α3β4 (b), α2β4(c), α2β2 (d), α1β1δε (e), α4α4 (f), α7 (g), α9β10 (h), α3β2 (i), and α4β2 (j) nAChRs. In each panel, “C” indicates the control response to ACh. *Xenopus laevis* oocytes expressing the indicated nAChRs were voltage-clamped at a holding potential of −70 mV. Representative ACh-evoked currents were obtained in the presence of 10 μM TxID-F. The oocyte was exposed to 10 μM TxID-F for 5 min (arrow), and we applied 1 s pulses of ACh to the oocyte in 1 min sweep; the flow rate of ND96 solution was 2 mL/min. All receptors were of rat origin, except α1β1δε, which was of mouse origin.
