# Supplementary material for: Fluorescently Labeled α-Conotoxin TxID, a New Probe for α3β4 Neuronal Nicotinic Acetylcholine Receptors
Source: Mar Drugs. 2022 Aug 12;20(8):511. doi: 10.3390/md20080511 (PMC9410468; doi:10.3390/md20080511)
Supplement: Supplementary file 1 [file marinedrugs-20-00511-s001.zip › marinedrugs-1816617-supplementary.pdf]

Supplementary Materials:

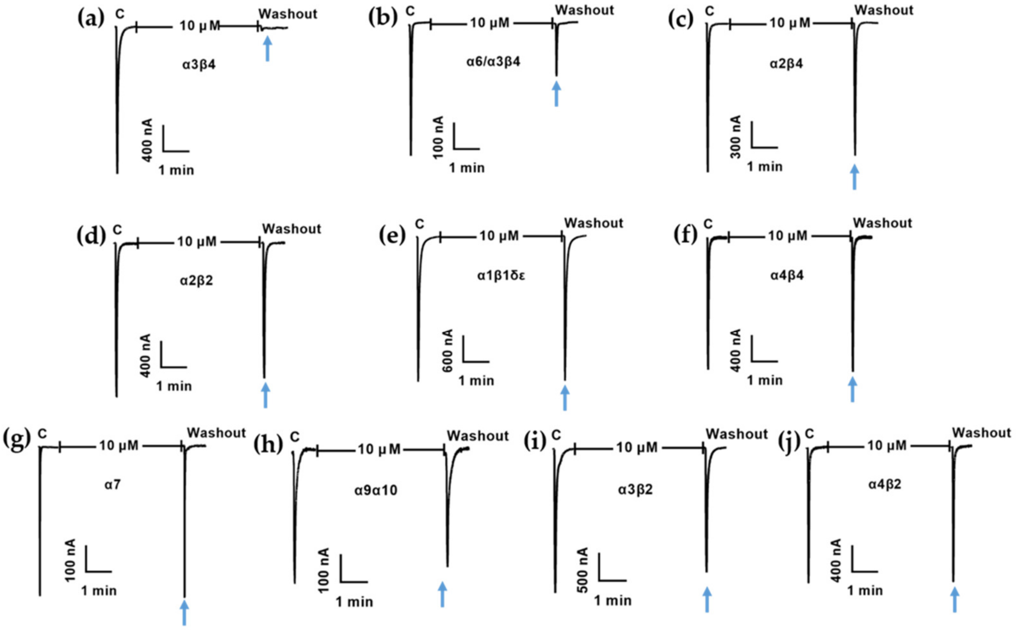

Figure S1: Electrophysiological activity of TxID-F. The potency of TxID-F on various nAChR subtypes. α3β4 (**a**), α6/α3β4 (**b**), α2β4(**c**), α2β2 (**d**), α1β1δε (**e**), α4α4 (**f**), α7 (**g**), α9β10 (**h**), α3β2 (**i**), and α4α2 (**j**) nAChRs. In each panel, "C" indicates the control response to ACh. *Xenopus laevis* oocytes expressing the indicated nAChRs were voltage-clamped at a holding potential of −70 mV. representative ACh-evoked currents were obtained in the presence of 10 μM TxID-F, the oocyte was exposed to 10 μM TxID-F for 5 min (arrow) and applied 1 s pulses of ACh to the oocyte in 1 min sweep, the flow rate of ND96 solution was 2 mL/min. All receptors are of rat origin, except α1β1δε which is of mouse origin.
